# An oral history of health psychology in the UK

**DOI:** 10.1111/bjhp.12418

**Published:** 2020-04-20

**Authors:** Francis Quinn, Angel Chater, Val Morrison

**Affiliations:** ^1^ School of Applied Social Studies Robert Gordon University Aberdeen UK; ^2^ School of Sport Science and Physical Activity University of Bedfordshire Bedford UK; ^3^ School of Psychology Bangor University UK

**Keywords:** history of health psychology, history of psychology, oral history

## Abstract

**Purpose**

An oral history of the development of health psychology in the United Kingdom.

**Methods**

Standard oral history methods produced interviews with 53 UK health psychologists, averaging 92 min in length. All interviewees entered the field from the 1970s to the 2000s, representing all four countries in the United Kingdom. A reconstructive mode of analysis, along with the few existing sources, was used to create a narrative of the history of health psychology in the United Kingdom. Audio recordings and transcripts will be archived for use by future researchers.

**Findings**

In the 1970s, medical schools in London recruited psychologists to teach, while also conducting pragmatic research on issues in healthcare. At the same time, some clinical psychologists began to work with physical health conditions in general hospitals. Partly influenced by developments in the United States and Europe, an identity of ‘health psychology’ developed and spread to researchers and practitioners doing work in psychology and health. In the 1980s, the field continued to attract researchers, including social psychologists working with health behaviours and outcomes, and clinical psychologists working in health care settings. During this time, it became formalized as a scientific field with the creation of the BPS Health Psychology Section, courses, and journals. In the 1990s, the field moved towards professional practice, which was controversial with other BPS divisions. However, it continued to grow and develop through the 2000s and 2010s.

**Conclusion**

Reflections on the development of UK health psychology represent the first historical narrative produced from oral testimony of those who were present at the time.

**Statement of Contribution**

***What is already known on this subject?***

Health psychology emerged in the 1970s, initially in the United States following an APA Task Force report. It developed from a range of precursor movements including psychosomatic medicine, while in the United Kingdom medical psychology was an additional precursor. The development of health psychology has been discussed for a range of countries including the United States and others, but historical scholarship relating to the United Kingdom has been limited.

***What does this study add?***

- From an oral history project, a narrative of UK health psychology’s development is built up.
- Influences included opportunities at medical schools from the 1970s onward.
- Growing interest in health behaviours as a test of social psychology theory was important.
- The experiences of clinical psychologists working in health care settings are demonstrated.
- Multidisciplinary influences on the emergence and shaping of health psychology are evident.

## Background

The history of psychology has been discussed in many texts over the years. However, relatively few (e.g. Lubek & Murray, 2018) focus on the history of health psychology. There are none that tell the story of how health psychology developed in the United Kingdom from those who formed part of that history. It was the aim of this project to produce such a narrative.

Science and practice that resembled health psychology took place throughout the 20th century in several countries, but not under that label until the 1970s. Indeed, ideas about mind–body interactions date back to ancient times. By the 19th century, medical writers in Europe and North America claimed that psychological factors influenced health. In the early 20th century, these ideas combined with psychology and psychoanalysis, leading to a multidisciplinary, international movement known as *psychosomatic medicine.* Then in the 1970s, *behavioural medicine* and *health psychology* appeared, initially in the United States (Belar, McIntyre & Matarazzo, 2012; Chater & Cook, 2014; Murray, 2014a; Pickren & Degni, 2011; Stam, 2014).

While historical scholarship of UK health psychology’s development is limited, Murray (2018) discusses its ‘pre‐history’, including *medical psychology*, strongly psychodynamic, as a precursor to health psychology. Johnston, Weinman and Chater (2011) provided a brief reminiscence‐based overview since the 1970s, Chater and Cook (2014) document the development from other disciplines and approaches, and Bennett (2015) has produced a history of UK clinical health psychology. Meanwhile, detailed histories of UK educational psychology (Arnold & Hardy, 2013) and clinical psychology (Hall, Pilgrim & Turpin, 2015) have appeared in recent years.

To address this gap in the literature, this oral history reconstructs the development of UK Health Psychology using oral testimony from those who were there, alongside the few published sources. With health psychology’s origins in the 1970s and 80s still in living memory, but with many of the first generation having retired, there was a golden opportunity to record oral history interviews for posterity and write a history providing greater depth and breadth of perspectives.

Good histories understand the past through the knowledge and values of that era, rather than the perspective of the present. They balance a naturalistic and personalistic approach (i.e., events as partly shaped by zeitgeist and partly by individuals’ actions). Finally, they balance external and internal history, incorporating developments within the field as well as outside influences (Furumoto, 1989). We aimed for a balanced approach: neither a ‘celebratory’ nor a ‘critical’ history but a balanced narrative. This article gives a brief overview of our findings and will form the first of a series of forthcoming articles that will explore specific developments.

## Method

We used standard oral history methods (Quinn, Chater & Morrison, 2020; Thompson & Bornat, 2017; Yow, 2014), using semi‐structured interviews.

### Participants

Oral history requires an informative sample with relevant first‐hand experiences. We identified potential interviewees from our knowledge of key contributors to the field in the 1970s–80s, as well as past chairs of the BPS Division of Health Psychology and its precursors, and added further names associated with later decades. We also asked interviewees for their suggestions.

Between July 2016 and November 2018, we approached 67 potential interviewees by email or letter; 53 agreed to interview (26 women, 27 men), with one joint interview. Most interviews were in person, usually at the interviewee’s home or office; eight were by Skype. UK nations of training or work (some more than one) broke down as England (45), Scotland (15), Wales (2), and Northern Ireland (4). Decade of first involvement with psychology as applied to health (paid work or postgraduate study) broke down as 1970s (12), 1980s (17), 1990s (21), and 2000s (3).

### Procedure

Ethical approval was granted by the School of Applied Social Studies ethics committee at the Robert Gordon University. After a mutually convenient date for interview was agreed, participants gave informed consent. Interviewing was shared between all authors, following an interview guide addressing relevant experiences and historical perspectives in a rough chronological order. Participants were also asked about influences on health psychology from within and outside the discipline, and perceptions of the field’s successes and limitations. Mean length of interview was 92 min (*SD* = 29). The 52 recordings total about 80 hr and the transcripts just over 550,000 words.

At the end of each interview, we invited interviewees to sign a copyright form allowing for their interview to be archived (all agreed). Interviewees were offered anonymity; however, none requested it (anonymity is discouraged in oral history; see Yow, 2014). Following verbatim transcription, we sent the transcript to the interviewee for edits or deletions. Any deletions were also made on the audio recordings. The edited version was used in the analysis and will be archived by the British Psychological Society’s History of Psychology Centre for future historical study once the current series is published.

### Analysis

To develop the narrative, a reconstructive mode of analysis was used, which is the most frequent in published oral history (Thompson & Bornat, 2017). This is not a formal analysis protocol as in qualitative research, but derives from the traditional scholarship of history. Listening to and reading the sources, the researchers identify patterns and develop a tentative understanding, which is supported, refuted, and refined using the sources. The researcher’s tentative understanding thus develops in a back‐and‐forth process.

## Findings

The interviewees’ experiences date from the 1970s. The narrative is structured by decade: 1970s (Applying Psychology to Medicine), 1980s (The New Frontier of Applied Psychology in the UK), 1990s (From Scientist‐Practitioners to Health Profession: Opportunities and Challenges), 2000s (Building a Discipline), and 2010s (Reflections and Future Direction).

### The 1970s: Applying psychology to medicine

The Todd Report (Royal Commission on Medical Education, 1968) broadened the UK medical curriculum to include social science, and medical schools in the 1970s increased the psychology they taught (see Griffiths, 1976). Some standalone medical schools in London recruited psychologists at this time to teach their students. John Weinman took a post at Guy’s Hospital Medical School in 1974 and recalled:

…I think they came to the conclusion that medical students needed to have a broader curriculum… to have teaching in things like psychology and sociology and ethics and communication skills and so on… a few medical schools offered these full‐time lectureships… we could more or less develop our own curriculum. (John Weinman)

Academic psychologists took up these lectureships, from backgrounds such as psychophysiology, social psychology, and cognition. Some had clinical psychology training, such as Marie Johnston, but they were primarily academic psychologists in academic posts. Few had pre‐existing expertise in health but they had scientific skills and often an interest in applied work. A few years before such a lectureship (see below), Marie Johnston took a post in 1971 at Oxford University (in the Health Services Evaluation Unit) that got her into health:

The project [statistician post] was about evaluating community hospitals, and I said I would take it on two conditions, one that they were randomly allocated, and two, that it influenced government policy…. I don’t think we influenced government policy, but that got me into health. That was when I got into health, but I hadn’t got the idea of health psychology. And I think the health psychology term only came in in about the mid‐70s’ (Marie Johnston)

Expansions in this curriculum allowed a slow expansion in psychology posts. These lectureships also provided time for research, medical collaborators, access to patients, students to help collect data, and interesting questions to pursue. Research funding was often available:

There was this slow trickle of doctors coming across asking questions like “well, you’re a psychologist, can you tell me why it is that some of my patients that don’t seem to be that ill, are coping so badly”… And these were doctors who could sort of see the bigger picture…. And so I started working with them… To be fair, it wasn’t the easiest environment to be in, it was a very biomedical environment, quite a lot of my more narrow biological colleagues were a bit dismissive of psychology. The students really liked it*.* (John Weinman)

This allowed a scientific literature to develop on psychology’s application to health and healthcare, such as preparation for surgery, and coping with illness and disability. This research was often pragmatic, quantitative, directly addressed issues seen in healthcare, used patients as participants, and at this time tended not to use much theory. Christine Eiser took up a research post on cognitive effects of radiotherapy at Great Ormond Street Hospital for Sick Children in 1975. She recalled that:

When I took the job they’d written the application very much in terms of IQ assessments, reading assessments, those kinds of things… The more I saw the children I felt it was the way families coped with the illness [leukaemia] that influenced how the children were… (Christine Eiser)

Psychophysiology had developed throughout the 20th century and by then had well‐formulated theory and methods. Such research at this time focused on stress, biofeedback, and other concepts which often had a clear relation to health. Derek Johnston recalls his post within a Medical Research Council Unit at Oxford University from 1970 to 1985, working on funded projects from ‘soft money’, which was seen as easier to secure at that time:

And to lower the heart rate and blood pressure essentially what people were trying to do was to relax and the biofeedback, if anything, was a distraction… I was going to physiology meetings and behaviour therapy meetings… I remained in behaviour therapy for a while rather than say health psychology. (Derek Johnston)

Addiction research also was also gaining momentum. Reflecting the mid‐20th century interest in behaviour as a cause of poor health, the Institute of Psychiatry in London developed an Addictions Research Unit, with smoking cessation as one focus. The unit employed psychologists of varying backgrounds, such as the social psychologist Richard Eiser in the mid‐1970s, and Robert West in the early 1980s. This unit provided an early health application for social psychology theories:

The main thing I was employed for was working with a psychiatrist called Mike Russell on smoking behaviour. And the sort of question was “well, you’re a social psychologist, you understand about attitude change, how do we change people’s attitude and behaviour towards smoking?” And I said I think that’s probably the wrong question. The question is probably more “why is it difficult to change people’s attitudes and behaviours?” (Richard Eiser)

This was on an MRC programme grant that was looking particularly at clinical interventions to help people stop smoking… (Robert West)

During the 1970s, clinical psychologists began to work in medical settings such as NHS general hospitals (Bennett, 2015) and applied behaviour therapy to problems of physical illness (Rachman, 1977; Rachman & Phillips, 1975). Early on, much of this work had a mental health focus, such as adjustment, or neuropsychology, often using behaviour therapy/modification, widely used then in British clinical psychology (Marks, 2015; Parry, 2015). But medical staff soon sought advice for non‐psychological health issues that were often behavioural in nature (e.g., medication adherence), and clinical psychologists began to move into patient work that would be seen as health psychology. Many also began to train hospital staff in how to manage some of these cases. At the general hospital in Dudley, West Midlands, at the end of the 1970s, Louise Wallace recalled that:

I fully recognised… there were very few psychologists but lots of psychology. And psychology could be done through other frontline staff if you taught them how to do it and supported them to do it… The idea that, if you like, you’re a consultant to the system rather than a hands‐on practitioner. (Louise Wallace)

Influences during this period came from the United States, Germany, and the Netherlands. Marie Johnston was an academic psychologist doing health research at Oxford University, who obtained clinical psychology training and in 1977 took up a lectureship at the Royal Free Hospital Medical School in London:

At that time [mid‐1970s] I was corresponding with people in America about the development of… a Task Force in Health Psychology, and that’s what went on to be [APA] Division 38… The conference [Boulder, USA] in 1974, George Stone was there and he basically laid out the agenda for health psychology… I came back totally inspired. (Marie Johnston)

The term ‘health psychology’ was not used during most of the 1970s. Terms such as ‘psychology applied to medicine’, ‘behaviour therapy ’, or ‘clinical psychology’ were used.

Nobody spoke the words ‘health psychology’ at all. And in fact, if you wanted to work in a health setting [as a practitioner] the only way to do so would be to have clinical [psychology] training, which was the reason I went in to get clinical training…there were no pathways as a psychologist unless you were clinical. (Lorraine Sherr)

### The 1980s: The new frontier of applied psychology in the UK

In the 1980s, the socio‐political climate shifted to the right, encouraging greater individual responsibility for health. A 1980 report on social inequality in health emphasized cultural and behavioural factors (Murray, 2018), and attempts at mass behaviour change characterized the UK Government’s response to HIV: ‘Don’t die of ignorance’. Health psychology’s emphasis on individual behaviour and response to illness fitted the zeitgeist of this period.

Academic and research posts and the identity of health psychology slowly grew, continuing the 1970s trend. The emerging health psychology revolved around a few centres, such as the London medical schools for research (e.g., Guy’s, Middlesex, and the Royal Free), while Gloucester and Dudley emerged as centres of health psychology practice in hospitals (still led by clinical psychologists), though practice also happened in other places.

On qualifying I gradually engineered myself out of psychiatry and into general practice… I started [doing health psychology] around 1984… I renamed myself…Health Psychologist. (Louise Earll)

Champions emerged who promoted health psychology as a scientific identity, and had mostly begun their health work in the 1970s, such as Marie Johnston and John Weinman. In practitioner work, champions also emerged in places with a supportive NHS management. They fought for opportunities and posts in the hospitals where they worked. Their success enabled more psychologists to join these centres, and these psychologists in turn created opportunities for others later. The Scientist‐Practitioner model during this period took a similar approach to today:

And one of the people on the interview panel [Royal Free] was Marie Johnston… Theresa Marteau was at the Royal Free too, and she was doing research looking at prenatal screening. And so I got involved with her research. And then Marie had a very great idea of bringing together the NHS and the academic bit… But I remember that when she was trying to set it up, she was trying to get people to come on board with health psychology, and I remember her saying to me ‘People don’t seem to be enthusiastic. Don’t understand what’s going on.’ And I said ‘Well, why don’t you tell people what health psychology is? Because I don’t know what health psychology is!’ (Susan Michie)

I worked clinically with the HIV patients at St Mary’s [Hospital in London] in the early days of the epidemic… really, conducted a lot of the HIV work in a research paradigm. And the reason behind that being that we would drive the practice by evidence of what works, what doesn’t work, what the nature of the problem was and how best to deal with it. And in a way that is the epitome of health psychology. (Lorraine Sherr)

During the 1980s, there was greater use of theory to explain patients’ behaviour and experience, mainly using American models. Leventhal, Nerenz and Steele's, (1984) common‐sense model was seen as especially helpful to explain coping with illness:

The psychology I was drawing upon were social psychologists like Lazarus and Folkman, looking at coping… There weren’t that many people in the UK working in this area. Having said that, there was Marie Johnston, she started by doing quite applied work in healthcare settings. Also at the time Jane Wardle and Andrew Steptoe and Derek Johnston as well. (Theresa Marteau)

Practitioners during this period (still clinical psychologists) often rejected a mental health approach and turned to health psychology to provide a body of theory that would be useful for their work. For example, Louise Earll approached Marie Johnston when ‘… I needed to acquire more academic rigour. It was clear that mental health models were not appropriate, the focus then was on abnormalities and deficits, problems and treatments’.

There was increasing research interest at this time in explaining unhealthy behaviours among well samples. Although there had been some use of health as an application of social psychology in Britain in the 1970s (e.g., Richard Eiser), the 1980s saw more social psychologists in the United Kingdom working with health‐related behaviours such as attendance at screening or smoking cessation. Theories were mostly American, such as the Health Belief Model (Rosenstock, 1974) and Theory of Reasoned Action (Fishbein & Ajzen, 1975). There was a growth in the study of addiction during the decade, which challenged a prevailing biomedical approach. According to Val Morrison, then working in Edinburgh:

People with drink and drug problems were either criminalised or they had a disease that had to be treated. What we [psychology] were trying to say was ‘yes, but there are social, contextual, emotional factors, beliefs and expectation factors that are influencing these behaviours’. It is easy to frame it now, but we were bringing a psychosocial model to the biomedical model of drug use. And I didn’t know about health psychology. I was an addictions researcher. (Val Morrison)

Many of the interviewees with a social psychology background gained their PhDs in the 1980s on topics not always related to health. Doctoral training continued in psychophysiology (e.g., stress and illness), and in a range of general psychology areas that had a health angle, such as disfigurement. Some interviewees became interested in health after the job search led to posts teaching medical or nursing students, or in postdoc positions with a health angle.

During my PhD [1982–1988] I think I had never heard of health psychology… I think I would have been an applied social psychologist… but very much applying psychology to health… coming across a new discipline of health psychology [at a conference in 1989] was quite exciting. (Paul Norman)

Other health psychology researchers took up traditional lectureships at universities, often but not always in psychology departments, training their own PhD students. A few lectured at polytechnics. This meant by the end of the 1980s the academic base no longer centred on London medical schools but had spread throughout the United Kingdom. More and more health‐related psychology work in research and practice by psychologists from various backgrounds was developing, united by a shared interest in health. Inevitably, they met informally and at conferences, and the label *‘health psychology’* was promoted by champions such as Marie Johnston as an umbrella term that united their work. Personal encounters at conferences with these champions could alter professional identities. Numerous interviewees described how they had been doing health psychology for years but had not identified themselves as such until they were introduced to the term:

When the division was formed in 1986 [as the BPS Health Psychology section], I looked back and thought, I have always been a health psychologist [nursing research, chronic pain work] and never known it! (Suzanne Skevington)

The label came to subsume work that pre‐dated it, such as the impact of stress on health. This re‐labelling of a psychologist’s activities as health psychology was facilitated by relationships (particularly meeting self‐identified health psychologists at conferences), informal contacts, mentoring, and being told or persuaded that one’s work was in fact ‘health psychology’. Textbooks (then mainly American) were published, highlighting this new field of health psychology, which aimed to spread knowledge and excitement (Murray, 2014a). Numerous interviewees highlighted a sense of home for their work and mentioned the supportive and friendly interpersonal atmosphere within the nascent health psychology, especially at conferences. John Weinman recalled, about the first BPS Health Psychology Section conference:

We were very keen to have a number of invited speakers… ’87 I’m thinking was probably the first… And we didn’t know many of the people. I can remember Marie and I being very struck at the end of the day, we went ‘phew, that went alright.’ And this is a slightly odd thing to say, but what we said was ‘what a nice crowd of people they seem to be’. (John Weinman)

#### Formalizing health psychology in the 1980s

Informal networks also developed, such as in London the ‘Subcommittee of Psychology Applied to Medicine’ (SPAM). These networks reached beyond the United Kingdom. American psychologists had an influence through correspondence and visits, such as Howard Leventhal and Charles Spielberger, and interactions with European colleagues helped build momentum:

So this was a Monty Python era… we would meet from time to time. And apart from go for a drink and catch up, we would look at the courses that we were running, sometimes seminar series, and just really networking. That was a very interesting and strong group of psychologists. (Theresa Marteau)

We [SPAM group] began to find other people, and a few things happened. So we met Stan Maes in the Netherlands. (Marie Johnston)

There were quite strong groups emerging in the Netherlands… Stan Maes was one of the key figures and he had a strong working relationship with Marie so I can remember him being around for various meetings. (Theresa Marteau)

During the 1980s, there was no dedicated UK journal publishing health psychology work. UK health psychologists could publish in a health section of the *British Journal of Clinical Psychology,* in the new European journal *Psychology and Health* (first issue 1987) and in a range of multidisciplinary and medical journals.

Desire emerged for a formal network within the British Psychological Society (BPS), with its own mechanisms for dissemination. The American Psychological Association had created a Health Psychology Division in 1978 (Wallston, 1996). BPS subdivisions represented fields of scientific interest (‘sections’) or professional practice underpinned by a body of knowledge and skills (‘divisions’). In the mid‐1980s, the BPS Medical Psychology Section was being reconsidered (medical psychology was primarily psychodynamic and closely linked to psychotherapy; see Murray, 2018); Johnston *et al. *(2011) described this as an immediate spur to creating a BPS Health Psychology Section. The new section was founded in December 1986, the same year as the European Health Psychology Society (in which Marie Johnston was also involved).

Being part of the BPS structure shaped how health psychology developed in the United Kingdom, because it provided a framework and incentives to move from a ‘section’ to a ‘division’. This included chartership as a mark of status, dedicated training routes, and competencies for professional practice. The attractions of ‘division’ status led to debate over whether health psychology should develop as a science only, as professional practice (and in what form?) or both. Interviews show this as controversial.

In this development stage, Masters degrees in health psychology began to appear in the 1980s. The first was a shared initiative between health psychologists at some of the London medical schools. John Weinman, then at Guy’s Hospital Medical School, remembered that:

We were starting to talk about teaching MScs in Health Psychology… the conversation took place between Marie [Johnston] who was at the Royal Free [Medical School], and Stan Newman who was at the Middlesex [Hospital Medical School], and we decided to create a University of London MSc in Health Psychology… It was the first one [1987/88]. (John Weinman)

It was soon followed by MSc courses at City University, the University of Surrey and Middlesex Polytechnic (now Middlesex University). The 1980s MSc curriculum at this time was totally academic.

At that time [late 1980s]…there were Masters in Health Psychology, but you wouldn’t have called them training courses, they weren’t training people for a job, there was no recognition within the NHS that health psychologists existed… there were absolutely no clinical skills in there at all. (Paul Bennett)

As the 1980s drew to a close, health psychology in the UK had a scientific and practice identity that was increasingly recognized and identified with, although many in the field maintained another, earlier professional identity (e.g., social psychologist, clinical psychologist).

### The 1990s: From scientist‐practitioners to health profession: Opportunities and challenges

Growth continued in the 1990s. Health psychology as an identity continued to spread, encouraged by champions, as Ronan O’Carroll found in the mid‐1990s after researching liver transplant and psychological outcomes:

[Marie Johnston] spoke to me about a job in health psychology and I said ‘But I’m not a health psychologist’. And she said ‘But you are, that’s the stuff you’re doing’. And I had never labelled it as such, but Marie did. (Ronan O’Carroll)

However, old and new identities still co‐existed: ‘I did, and I still do, feel like I’m a social psychologist doing health stuff’. (Mark Conner)

#### Towards practice

In 1990, the BPS Health Psychology Section held a debate at Regent’s College in London and a vote at the 1991 annual conference. The choice was between remaining a science or also moving towards practice. The 1991 attendees voted ‘overwhelmingly’ for the latter (Bennett, 2015). This led to the section becoming a BPS Special Group in 1993, as a halfway house to ‘division’ status.

In preparation for becoming a BPS division, suitable competencies and training routes had to be developed. However, these had to be approved by a vote of representatives from all the BPS divisions. The Division of Clinical Psychology (DCP) opposed one‐to‐one clinical work by health psychologists. Clinical psychology in the United Kingdom had a history of boundary disputes over clinical roles, with psychiatry in the 1950s–60s, other mental health professions providing psychotherapy in the 1970s–80s, and counselling psychology in the early 1990s (Hall, 2015; Parry, 2015). The DCP’s opposition led to a compromise on training for health psychologists, focusing on consultancy, training health care professionals, and research as core competencies, with behaviour change as an option. The compromise of excluding one‐to‐one intervention allowed the Special Group to become the BPS Division of Health Psychology (DHP) in 1997. In parallel, a new subsection of the DCP (the Faculty of Clinical Health Psychology) was formed in December 1998 to represent clinical psychologists working in health care settings (Bennett, 2015). Clinical skills were introduced to the core curriculum in the 21st century, which was then accepted within the BPS.

What happened over time was that the nature of the interventions we could find ourselves doing as health psychologists became clearer, and it became clearer that that wasn’t really treading on the toes of clinical psychologists. (Mark Forshaw)

Practice in the 1990s was still mainly by clinical psychologists taking up local NHS opportunities where the climate was supportive. From the formation of the DHP in 1997, the first practitioners were trained as health psychologists (rather than as clinical psychologists working in health). Many with relevant experience ‘grand‐parented’ into full membership of the division and gained Chartered Health Psychologist status (sufficient to practice at that time with a practising certificate issued by the BPS). This route consisted of a variety of differing routes and experience and closed in the 2000s:

I suppose lots of us had done voluntary work or had done assistant things perhaps. Or even in our qualitative data collection, you end up learning how to communicate… So there would have been a variety of routes and different ways people would have got there. I don’t think there was a one size route. (Karen Rodham)

#### Health psychology research in the 1990s

Researchers continued to build the literature and founded new journals, including in 1996 both the *British Journal of Health Psychology* and the *Journal of Health Psychology*. Growth in student places at UK universities created more academic posts for researchers and PhD graduates, including at the new universities created in 1992 from the polytechnics. This growth saw some universities become major centres where health psychology had not previously been represented. By the end of the 1990s, health psychology was more widespread than ever in university departments of psychology, medicine, and health professions. At university psychology departments, health psychology was more often taught to undergraduates (sometimes billed as an application of social psychology). This influenced some of the 1990s generation of psychology graduates to enter postgraduate study specifically to become a health psychologist (rather than another branch of psychology such as social or clinical psychology).

Social psychology theories became prominent in health behaviour research in the 1990s, such as the Theory of Planned Behaviour (Ajzen, 1985). Rory O’Connor was a PhD student and later lecturer in the 1990s. He remarked that:

I think in the 1990s we were so fixated on the social cognitive models. Now, I think that was really important in some ways because it led to an enhanced understanding of the determinants of health behaviour. But the problem was, too many studies were cross‐sectional and it wasn’t often clear how you would translate this into saving lives or changing people’s behaviour. (Rory O’Connor)

Most health psychology research up to this period had been quantitative and from positivist epistemologies, with research and theory focused on individual determinants (e.g., cognitions). This was likely shaped by dominant ideas in the mainstream psychologies from which it drew, such as American social psychology and British clinical psychology. During the 1990s, some questioned the dominance of these methods in health psychology. One consequence was interest in qualitative research. Patrick Hill, a practitioner who later worked in government, remembered how in 1996:

Jonathan Smith published a cover article in *The Psychologist* [BPS magazine] about using IPA [interpretative phenomenological analysis]… and it was the first that I was aware of a mainstream publication of a qualitative piece of research… It sounds really basic now, but really it wasn’t something that was generally done even then. (Patrick Hill)

During the 1990s, calls grew for health psychology to be more informed by social, political, and cultural factors. This *critical health psychology* grew in the 1990s with publications voicing ideas previously on the sidelines, as part of a wider critical psychology movement (Murray, 2014b). It also advocated a wider range of research methods. David Marks started the *Journal of Health Psychology* in 1996:

…perhaps to go beyond the normal boundaries of what was seen as being established health psychology…human behaviour is economically and politically embedded. And…there are many ways to approach an understanding of psychological issues. And so therefore qualitative methods and other methods should be given equal standing as approaches to empirical work. (David Marks)

### The 2000s: Building a discipline

By the year 2000, health psychology in the United Kingdom had the common features of a science and health profession: a professional body, journals, conferences, textbooks, and training routes. It had subsumed research areas that pre‐dated it, such as stress, coping and disease and the psychology of pain. Methods and viewpoints were becoming more diverse. It sat within a range of departments, in universities and to a lesser extent in healthcare settings. Health psychologists in university posts and MSc Health Psychology courses continued to increase. Practitioners could now be trained as health psychologists without a clinical psychology background. However, many practitioners were clinical psychologists and health psychology practice overlapped with clinical health psychology (Bennett, 2015). UK critical health psychologists hosted a conference in Birmingham in 2001 at which the International Society of Critical Health Psychology formed (ISCHP, 2019), although critical health psychology remained outside the mainstream.

Focus continued to increase on health behaviour research, drawing primarily on the social cognition models that mostly derived from late 20th century American social psychology. Cross‐sectional survey designs were still frequently used to test these theories but as the 2000s wore on it became clear that the limits of such designs had been reached and more intervention (i.e., experimental) designs started to be used. Numerous theories were used, and not all researchers used social cognition models, but the Theory of Planned Behaviour (Ajzen, 1985) dominated for some time.

#### Health psychology and government

The 2000s marked the entry of health psychology into advising government. Arranged and part‐funded by the DHP, Charles Abraham and Susan Michie served a secondment to the UK Government’s Department of Health in 2002–03. They reviewed evidence for policy decisions and advised civil servants, ministerial advisers, and the committee producing the second Wanless report about future provision of NHS services (Wanless, 2004). They also contributed to the *Choosing Health* white paper (Department of Health, 2004). A further secondment followed, at the UK Government’s Health Improvement and Protection Directorate, by Susan Michie and Nicky Rumsey. Chris Armitage recalled his involvement during his term as DHP Chair:

One of the roles of DHP I think is to try and influence policy and make sure that health psychology is at the centre of any health policy or anything that’s to do with behaviour change and that kind of thing. And there’d been a proposal to have a one day a week post funded by the Division of Health Psychology to have someone actually working physically in London with the Faculty of Public Health. So one of the first things I did was on the interview panel for that post. And it ended up being a job‐share between Susan Michie and Nicky Rumsey. (Chris Armitage)

Susan and I worked on the NHS Health Trainer thing and really fought hard, with the Department of Health team, over a number of years to get that rolled out and adopted. And I think we were influential in winning hearts and minds in order to do that. And then [2010] there was a change of government and then the whole division got wiped out. (Nicky Rumsey)

By this time, devolved governments in Scotland, Wales, and Northern Ireland controlled most internal affairs. Diane Dixon and Marie Johnston recalled how DHP Scotland persuaded DHP to part‐fund a secondment to the Scottish Government’s health directorate in 2007. They were selected from a competitive process and, for a few days per month, advised civil servants on health psychology‐related evidence. This led to the development of a set of competencies needed for health behaviour change (Dixon & Johnston, 2010):

So the questions that government puts to you pushes you to develop things… Diane [Dixon] and I were asked ‘How can we take someone who is trained to change smoking behaviour to change dietary behaviour? What are the shared competencies? (Marie Johnston)

The mid‐2000s were a time of preparation for statutory regulation of practitioner psychologists. In 2009, the UK Government’s Health Professions Council (HPC: now Health and Care Professions Council, HCPC) took over the regulation of the practice of psychology (from the BPS), and ‘health psychologist’ became a legally protected title. Daryl O’Connor was DHP chair in 2006–7, and Angel Chater sat on the DHP committee; they recalled a busy time updating the curriculum for HPC approval:

Statutory regulation was huge. What would happen when the BPS stopped becoming the regulators of the practicing certificate, and the HPC were going to take them on board? So there were lots of conversations about our skills, what would the training look like, if the training needed to be redeveloped? (Angel Chater)

But then we had all the stuff about the levels of thresholds and the standards of training. So a lot of work we were doing at that stage… was to get agreed through the Health Professions Council, as it was then, to agree to the level of training… But certainly we got what we wanted… (Daryl O’Connor)

Changes were made to the 1990s curriculum that had been created to meet the needs of BPS Chartership, adding one‐to‐one intervention competencies for HPC registration. Despite opposition to such competencies in the 1990s by the Division of Clinical Psychology, this addition to the training skill set was unopposed.

However, the training was still self‐funded. Several DHP chairs and committee members met with policymakers about government‐funded places, often perceiving progress but never success. Claire Hallas and Sasha Cain, then on the DHP committee, recalled how by 2010:

Hallas: ‘We got into meetings with the workforce development confederations, we were round the table with the senior clinicians, the senior policy makers, the Department of Health people. And probably our legacy that no one will ever know about is that we got…’

Cain: ‘I think it was 120’

Hallas: ‘120 agreed training places for health psychology at that meeting’.

Interviewer: And what happened to those training places?

Cain: ‘They never happened’.

Hallas: ‘The government collapsed about two weeks later’.

Nevertheless, DHP Scotland achieved some funding success in the mid‐2000s. Training of psychologists for NHS Scotland was funded by NHS Education for Scotland (NES). Its head of psychology training was Ann Smyth, a clinical psychologist, who championed funded Stage 2 training for health psychologists with Vivien Swanson and Ronan O’Carroll. The result was approximately four funded 2‐year trainee posts each year from 2007, working on regional health board objectives:

We [Ann Smyth, Beth Alder, Ronan O’Carroll] had discussions with NES about whether they could offer some support… So we had about two years of exchanging documents, conversations, trying to convince people that that was a good thing to fund. (Vivien Swanson)

### The 2010s: Reflections and future directions

By the 2010s, there was a shift from predicting health behaviour using dominant theories from social psychology, towards health behaviour change. This matched the zeitgeist in public discourse on health but the dominance of health behaviour research was noted:

I think, looking back [to early 2000s], there was a more even split in those days. I think the behaviour change work really took off and took the ascendancy over recent years. (Alison Wearden)

Developments in the 2010s included gaining a role for health psychology in healthcare research funding with seats on grant committees (e.g., National Institute for Health Research), and increasingly, a health psychologist was expected on research teams where there was a behavioural angle. A focus on applied science had been a consistent theme throughout UK health psychology’s development:

So, I absolutely agree that the core of health psychology is as I think it was in the beginning, applying the science and developing the science. (Jo Hart)

The practice of health psychology continued to mature. As new entrants came into the profession via the standard training routes, fewer had clinical psychology backgrounds, and clinical health psychology has become a parallel profession, sometimes in competition for NHS posts. However, NHS posts for which health psychologists are eligible have not increased to the level previously hoped. These later developments of health psychology’s history will feature in future publications.

## Discussion

Health psychology, as Ebbinghaus (1908) famously described psychology itself, has a long past but a short history. In the United Kingdom, it is an identity that united disparate activities of practice and research, subsuming the psychology work in health and illness that pre‐dated it. Many interviewees described serendipity as what brought them to health psychology, even in the 1990s, often through an unexpected opportunity such as a chance meeting, a job advert or advertised PhD. These were sometimes driven by a question that needed answering from another profession, such as medicine. However, not all interviewees embraced the identity of health psychologist. Health psychology seems not just an identity but also an enterprise to which psychologists of any identity can contribute. Since the 1990s health psychology in the United Kingdom has become more pluralistic, but some perspectives are still outside the mainstream.

### Limitations

Just as a single piece of psychological research does not provide unquestioned truth, nor does a history (Goodwin, 2015). Differing historiographical methods may produce different histories (Lubek & Murray, 2018). Indeed, historical scholarship often produces competing interpretations, which may be refined as new sources are explored (Claus & Marriott, 2012). We were not able to interview everyone that we hoped, there may be people we did not identify with important experiences, and some groups are not as numerous in our sample (e.g., critical health psychology, clinical health psychology, interviewees in Wales and Northern Ireland). In addition, we did not analyse publications nor documentary sources such as committee meeting minutes. Future scholars should explore the archived interviews, record further interviews, explore other relevant sources, and refine our interpretations.

### Conclusion

While history is shaped by the zeitgeist of the day, its direction is also shaped by individual and group action. Our choices today in our science, practice, teaching, advocacy, and leadership will shape the health psychology that we hand to the generations that follow us, and our impact on the health of the nation.

## Conflicts of interest

All authors declare no conflict of interest.

## Authors’ contributions

FQ and AC conceived the project idea and were successful in gaining funding from the Royal Society. FQ, AC, and VM (with input from TC) developed the interview strategy and list of interviewees. All authors conducted a number of interviews, contributed to the development of the narrative, contributed to the drafting of the manuscript, and have given approval for submission.

## Data Availability

The interviews and transcripts created as part of this oral history project, subject to interviewee’s consent, will be archived at the British Psychological Society’s History of Psychology Centre (https://www.bps.org.uk/hopc) following the publication of our final paper in this series.
